# Evaluation of the 3-Minute Diagnostic Confusion Assessment Method for Identification of Postoperative Delirium in Older Patients

**DOI:** 10.1001/jamanetworkopen.2021.37267

**Published:** 2021-12-13

**Authors:** Jordan Oberhaus, Wei Wang, Angela M. Mickle, Jennifer Becker, Catherine Tedeschi, Hannah R. Maybrier, Ravi T. Upadhyayula, Maxwell R. Muench, Nan Lin, Eva M. Schmitt, Sharon K. Inouye, Michael S. Avidan

**Affiliations:** 1 Department of Anesthesiology, Washington University in St Louis, St Louis, Missouri; 2 Department of Mathematics and Statistics, Washington University in St Louis, St Louis, Missouri; 3 Division of Biostatistics, Washington University in St Louis, St Louis, Missouri; 4 Aging Brain Center, Institute for Aging Research, Hebrew Senior Life, Boston, Massachusetts

## Abstract

**Importance:**

Delirium is a common postoperative complication in older patients that often goes undetected and might lead to worse outcomes. The 3-Minute Diagnostic Confusion Assessment Method (3D-CAM) might be a practical tool for routine clinical diagnosis of delirium.

**Objective:**

To assess the 3D-CAM for detecting postoperative delirium compared with the long-form CAM used for research purposes.

**Design, Setting, and Participants:**

This cohort study of older patients enrolled in ongoing clinical trials between 2015 and 2018 was conducted at a single tertiary US hospital. Included participants were aged 60 years or older undergoing major elective surgical procedures that required at least a 2-day hospital stay. Data were analyzed between February and April 2019.

**Exposures:**

Surgical procedures of at least 2 hours in length requiring general anesthesia with planned extubation.

**Main Outcomes and Measures:**

Patients were concurrently assessed for delirium using the 3D-CAM assessment and the long-form CAM, scored based on a standardized cognitive assessment. Agreement between these 2 methods was tested using Cohen κ with repeated measures, a generalized linear mixed-effects model, and Bland-Altman analysis.

**Results:**

Sixteen raters conducted 471 concurrent CAM and 3D-CAM interviews including 299 patients (mean [SD] age, 69 [6.5] years), the majority of whom were men (152 [50.8%]), were White (263 [88.0%]), and had noncardiac operations (211 [70.6%]). Both instruments had good intraclass correlation (0.84 for the CAM and 0.98 for the 3D-CAM). Cohen κ demonstrated good overall agreement between the CAM and 3D-CAM (κ = 0.71; 95% CI, 0.58 to 0.83). According to the mixed-effects model, there was statistically significant disagreement between the 3D-CAM and CAM (estimated difference in fixed effect, −0.68; 95% CI, −1.32 to −0.05; *P* = .04). Bland-Altman analysis showed the probability of a delirium diagnosis with the 3D-CAM was more than twice the probability of a delirium diagnosis with the CAM (probability ratio, 2.78; 95% CI, 2.44 to 3.23).

**Conclusions and Relevance:**

The 3D-CAM instrument demonstrated agreement with the long-form CAM and might provide a pragmatic and sensitive clinical tool for detecting postoperative delirium, with the caveat that the 3D-CAM might overdiagnose delirium.

## Key Points

**Question:**

Can the 3-Minute Diagnostic Confusion Assessment Method provide similar delirium detection as the standard Confusion Assessment Method for older patients who have undergone surgery?

**Findings:**

In this cohort study of 299 patients aged 60 years or older who had undergone surgery, the 3-Minute Diagnostic Confusion Assessment Method showed good agreement with the longer Confusion Assessment Method.

**Meaning:**

These results suggest the 3-Minute Diagnostic Confusion Assessment Method might be a useful tool for clinical delirium detection in patients who have undergone surgery.

## Teaser

This cohort study evaluates the performance of the 3-Minute Diagnostic Confusion Assessment Method (3D-CAM) for detecting postoperative delirium in older patients.

## Introduction

Delirium is an acute and fluctuating change in mental status, including inattention, disorganized thinking, and altered level of consciousness.^1^ Delirium is common in older patients following surgical procedures, especially those requiring intensive care unit (ICU) stays.^2^ Delirium has been associated with increased morbidity, mortality, likelihood of institutionalization, and length of hospital stay.^3,4,5,6^ Delirium is often not diagnosed, and this important gap in clinical practice is at least in part due to a lack of validated, practical screening tools. It is possible that improving delirium detection would help clinicians to implement early interventions for vulnerable patients, potentially averting negative outcomes.

One of the most commonly used and validated instruments for delirium detection is the Confusion Assessment Method (CAM).^7^ The CAM identifies delirium by the presence of 4 cardinal features scored through a brief cognitive assessment: (1) acute change and fluctuating course, (2) inattention, (3) disorganized thinking, and/or (4) altered level of consciousness. The 3-Minute Diagnostic Interview for Confusion Assessment Method (3D-CAM) was derived from the CAM with the goal of creating an abbreviated tool to identify delirium^8^ that required less extensive training. As a screening tool, the 3D-CAM was designed to maximize sensitivity so that cases of potential delirium would not be missed. The 3D-CAM takes less than 3 minutes to administer and has the potential to be implemented as part of routine clinical care. The aim of this study was to assess the agreement of the 3D-CAM with the long-form CAM for identification of delirium in older adults following major surgical procedures at a single center.

## Methods

This manuscript complies with the Strengthening the Reporting of Observational Studies in Epidemiology (STROBE) reporting guideline for observational studies. Patients were enrolled in the Prevention of Delirium and Complications Associated with Surgical Treatments (PODCAST) trial,^9,10^ the Electroencephalography Guidance of Anesthesia to Alleviate Geriatric Syndromes (ENGAGES) trial,^11,12^ and/or the Systematic Assessment and Targeted Improvement of Services Following Yearlong Surgical Outcomes Surveys (SATISFY-SOS) study.^13^ All patients were aged 60 years or older and underwent major elective surgical procedures at Barnes Jewish Hospital in St Louis, Missouri. The PODCAST^9,10^ and ENGAGES^11,12^ trials were ongoing randomized clinical trials examining respectively the effectiveness of subanesthetic ketamine and electroencephalographic guidance of anesthesia at decreasing postoperative delirium incidence. Patients in these studies were assessed at least daily for delirium up to postoperative day 5. SATISFY-SOS is an ongoing registry that assesses patient-reported outcomes following an operation. This study was conducted under the institutional review board approvals of the 3 parent studies from the Washington University School of Medicine, and all patients provided written informed consent. Patients were included in this substudy regardless of their group assignment in the 2 randomized trials.

Investigators were rigorously trained in the use of the CAM and 3D-CAM instruments. The training protocol for the CAM interview has previously been described.^10,11,14,15^ In brief, it consisted of an initial 3-hour instructional session on the conduct and scoring of the CAM. This included review of standardized videos, where trainees would watch a prerecorded interview by an experienced rater (defined as someone who had previously completed the full-day CAM training program as led by the creator of the CAM or completed the training protocol). After video scoring accuracy was determined by an experienced rater, the trainee would then observe an experienced rater conducting a CAM interview. Subsequently, the trainee and the experienced rater would score the CAM independently. Once the trainee and experienced rater agreed on all 12 features of the CAM for 2 patients with delirium and 2 patients without, the trainee was then observed by the experienced rater as they conducted interviews. After being observed for 2 interviews, and with approval from the experienced rater, the trainee was considered eligible to independently conduct CAM assessments. Additional 3D-CAM training consisted of a standard series of video interviews available at the Hospital Elder Life Program website.^16^ After watching the video interviews, investigators had to agree on 2 patients with delirium and 2 patients without based on 3D-CAM determinations before assessing patients for delirium using the 3D-CAM instrument.

For the purpose of this study, the CAM was rearranged so that completion of the 3D-CAM questions would occur first. Both the CAM and 3D-CAM assessor approached the patient together. The CAM assessor conducted the interview, and the 3D-CAM assessor collected patient responses to the 3D-CAM questions in parallel while observing the CAM interview. Once the 3D-CAM questions were completed in the context of the interview, the 3D-CAM assessor would exit the room. This allowed the 3D-CAM questions to be complete but allowed masking of the 3D-CAM assessor to the additional information collected for the CAM (ie, extended patient-reported delirium symptoms, delusions, disorientation, disturbance of sleep, digits forward, and memory impairment). Additionally, the 3D-CAM has 2 questions to ask family members whether they have noticed a change in the patient’s mentation from baseline. Family members, or the bedside nurse in the absence of family members, were asked these questions without the CAM assessor present. The CAM and 3D-CAM assessors independently scored their respective assessments, masked to the other’s scoring. The time required to complete each assessment, excluding scoring time, was also documented.

Patients were assessed by the paired raters daily until follow-up was completed per the relevant study protocol or patients were nondelirious on 3 consecutive interviews as determined by the CAM. CAM and 3D-CAM pairs completed on postoperative day 0 were conducted at least 2 hours after the end of anesthesia care. Patients enrolled in the PODCAST study had CAM and 3D-CAM assessments both in the morning and afternoon. Those in the ENGAGES and SATISFY-SOS studies had assessments completed only in the afternoon.

### Statistical Analysis

We previously published a detailed description of the statistical methods that we used in this study.^17^ Briefly, a generalized linear mixed model (GLMM) was used for interrater reliability as well as method agreement (CAM vs 3D-CAM). Even though only 1 CAM and 3D-CAM were conducted at any given interview (ie, 1 rater used the CAM and 1 the 3D-CAM), the GLMM method is able to provide an estimate of interrater reliability for each instrument. The extent of agreement between the 2 instruments was assessed, with appropriate adjustment for multiple delirium assessments in individual patients using a Bland-Altman analysis as well as Cohen κ. In addition to the agreement on the overall presence or absence of delirium, presence or absence of the 4 cardinal features of delirium (ie, acute change and fluctuating course, inattention, disorganized thinking, and altered level of consciousness) were tested post hoc using the same statistical methodology to assess where there was most discordance and concordance in the scoring algorithms of the 2 instruments. Data analysis was completed using SAS version 9.4 (SAS Institute) as well as R version 3.4.2 (R Project for Statistical Computing). The statistical significance level for all analyses including the GLMM was specified by convention as α = .05, and results were presented with 95% CIs. Cohen κ results were interpreted by Landis and Koch’s guidelines,^18^ which characterize κ values over 0.75 as substantial.

## Results

A total of 299 patients had 471 concurrent assessments at different time points (Table). The mean (SD) age of patients was 69 (6.5) years, 152 (50.8%) were men, and 263 (88.0%) were White. Most patients were undergoing noncardiac operations (211 [70.6%]) and were not cognitively impaired (Short Blessed Test median [IQR] score, 4 [0-5]; 8-item Interview to Differentiate Aging and Dementia median [IQR] score, 0 [0-1]). The median (IQR) time spent on conducting assessments with each patient was 3 minutes (2-4 minutes) for the 3D-CAM and 8 minutes (6-10 minutes) for CAM (*P* < .001). These times do not include the time taken for scoring the assessments. Sixteen different raters participated in patient interviews.

**Table.** Patient Characteristics

Patient characteristics	Patients, No. (%) (N = 299)
Age, mean (SD), y	69 (6.5)
Sex	
Women	147 (49.2)
Men	152 (50.8)
Noncardiac surgical procedure	211 (70.6)
Race	
African American	21 (7.0)
White	263 (88.0)
Other or unknown^a^	15 (5.0)
ASA status^b^	
1	2 (0.7)
2	47 (15.7)
3	162 (54.2)
4	88 (29.4)
No. of comorbidities	
0	53 (17.7)
1	38 (12.7)
2	66 (22.1)
≥3	142 (47.5)
History of alcohol use^c^	152 (50.8)
History of tobacco use^d^	187 (62.5)
High risk for obstructive sleep apnea^e^	109 (36.5)
Hearing impairment^f^	58 (20.1)
Vision impairment^f^	127 (44.3)
Barthel Activities of Daily Living Index, median (IQR)^f^^,^^g^	100 (100-100)
8-item Interview to Differentiate Aging and Dementia, median (IQR)^f^^,^^h^	0 (0-1)
Short Blessed Test for Cognition, median (IQR)^f^^,^^i^	4 (0-5)

Abbreviation: ASA, American Society of Anesthesiologists.

^a^ Other races included American Indian and Alaska Native, Asian, Native Hawaiian or other Pacific Islander, or chose not to report.

^b^ ASA physical status classification system uses the following categories: 1, healthy patient; 2, mild systemic disease; 3, severe systemic disease; and 4, severe systemic disease that is a constant threat to life.

^c^ Alcohol consumption was obtained from patients’ medical health records.

^d^ Tobacco use was obtained from patients’ medical health records.

^e^ High-risk obstructive sleep apnea is defined as a score of 5 or higher on the STOP-BANG sleep apnea questionnaire (snoring history, tired during the day, observed stop breathing while sleeping, high blood pressure, body mass index >35 [calculated as weight in kilograms divided by height in meters squared], age >50 years, neck circumference >40 cm, and male gender).

^f^ The following patient characteristics had data missing (with patient totals listed): hearing impairment (288 patients), vision impairment (287 patients), Barthel Activities of Daily Living Index (233 patients), 8-item Interview to Differentiate Aging and Dementia (238 patients), and Short Blessed Test for Cognition (239 patients).

^g^ Barthel Activities of Daily Living Index is scored on a 100-point scale categorized as follows: less than 20, totally dependent; 20 to 39, very dependent; 40 to 59, partially dependent; 60 to 79, minimally dependent; and 80 to 100, independent.

^h^ The 8-item Interview to Differentiate Aging and Dementia scale is 0 to 1, normal cognition; 2 or greater, cognitive impairment is likely to be present.

^i^ Short Blessed Test for Cognition is rated on a 4-point scale, with 0 to 4 indicating normal cognition; 5 to 9, questionable impairment; and 10 or greater, impairment consistent with dementia.

While testing for interrater reliability, the GLMM returned intraclass correlation values for proportion of variation by patients of 0.84 for the CAM and 0.98 for the 3D-CAM. These correlation values denote a large amount of patient variation with a small variation owing to the raters. Therefore, the raters had good agreement among themselves using both instruments (ie, the instruments demonstrated good interrater reliability).

Method agreement between the 3D-CAM and CAM was then tested and found to be significantly different by GLMM (estimated difference in fixed effect, −0.68; 95% CI, −1.32 to −0.05; *P* = .04). Therefore, the CAM and the 3D-CAM demonstrated method disagreement. Further tests of agreement for each of the 4 cardinal features was also tested using the GLMM. Agreement between these features was found to be significantly different for acute change (estimated difference in fixed effect, 1.23; 95% CI, 0.71 to 1.74; *P* < .001), inattention (estimated difference in fixed effect, −0.84; 95% CI, −1.03 to −0.65; *P* < .001), and disorganized thinking (estimated difference in fixed effect, −1.48; 95% CI, −2.04 to −0.93; *P* < .001), while altered level of consciousness was found not to be significantly different (estimated difference in fixed effect, 0.66; 95% CI, −0.13 to 1.45; *P* = .09).

**Figure 1.**
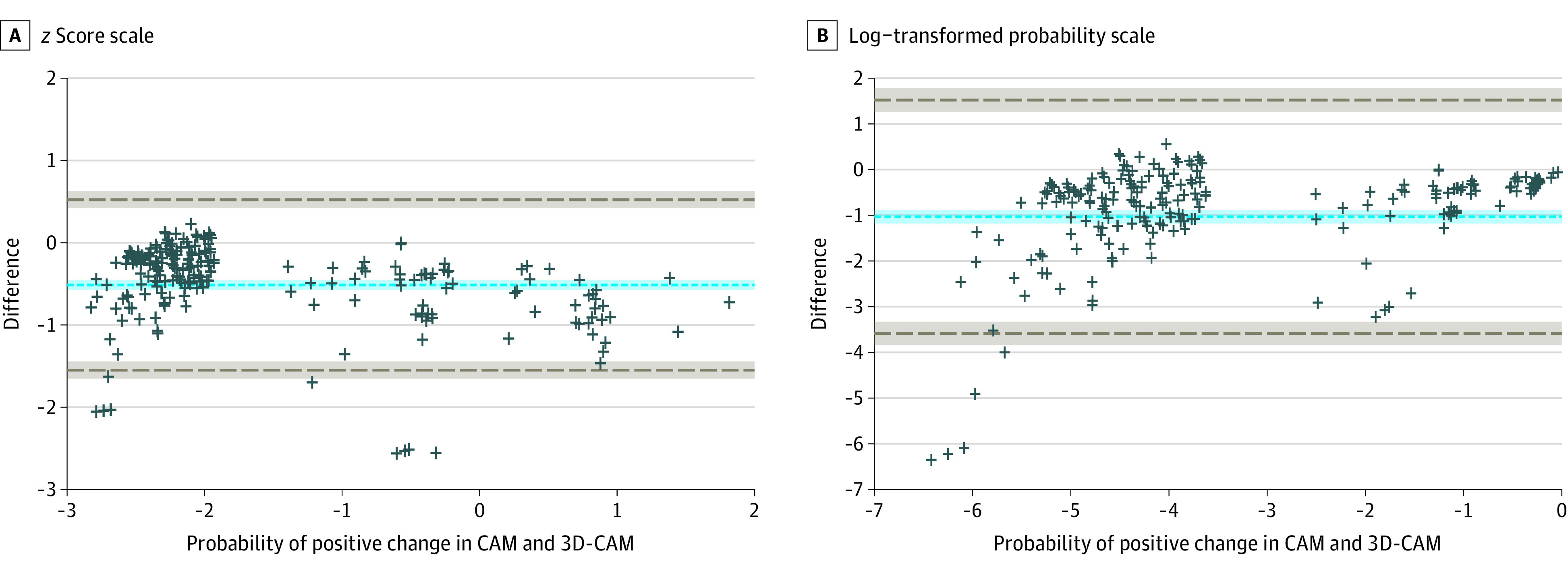
Bland-Altman Plots for the 3-Minute Diagnostic Confusion Assessment Method (3D-CAM) and Long-Form CAM Instruments Each plus sign represents 1 patient (299 total patients); dashed gray lines, 95% agreement limits; and the dashed blue line, the mean difference.

An individual-level summary measure for each method was given based upon the latent variable formulation of the GLMM used for testing method agreement.^17^ That is, a pair of model-estimated continuous delirium outcomes for the CAM and the 3D-CAM was determined for each of the 299 patients and used to plot the Bland-Altman diagram. A pair of model-estimated binary delirium outcomes was then generated based on the latent variable for the evaluation of Cohen κ. The Bland-Altman analysis provided a visual representation of agreement, as well as agreement in terms of probability. This method plots the observations with the average of the outcomes ([Outcome A + Outcome B]/2)) on the x-axis and the difference between the 2 paired measurements (Outcome A − Outcome B) on the y-axis. Using the mean difference between the 2 instruments, a calculation can determine the probability of a positive CAM compared with the 3D-CAM. The mean difference of the Bland-Altman on the log scale was −1.03 (95% CI, −1.18 to −0.88) (Figure 1). Therefore, the probability of a positive CAM was 0.36 (95% CI, 0.31 to 0.41) times the probability of the 3D-CAM, the inverse of which shows that a positive 3D-CAM was 2.78 (95% CI, 2.44 to 3.23) times the probability of a positive CAM.

**Figure 2.**
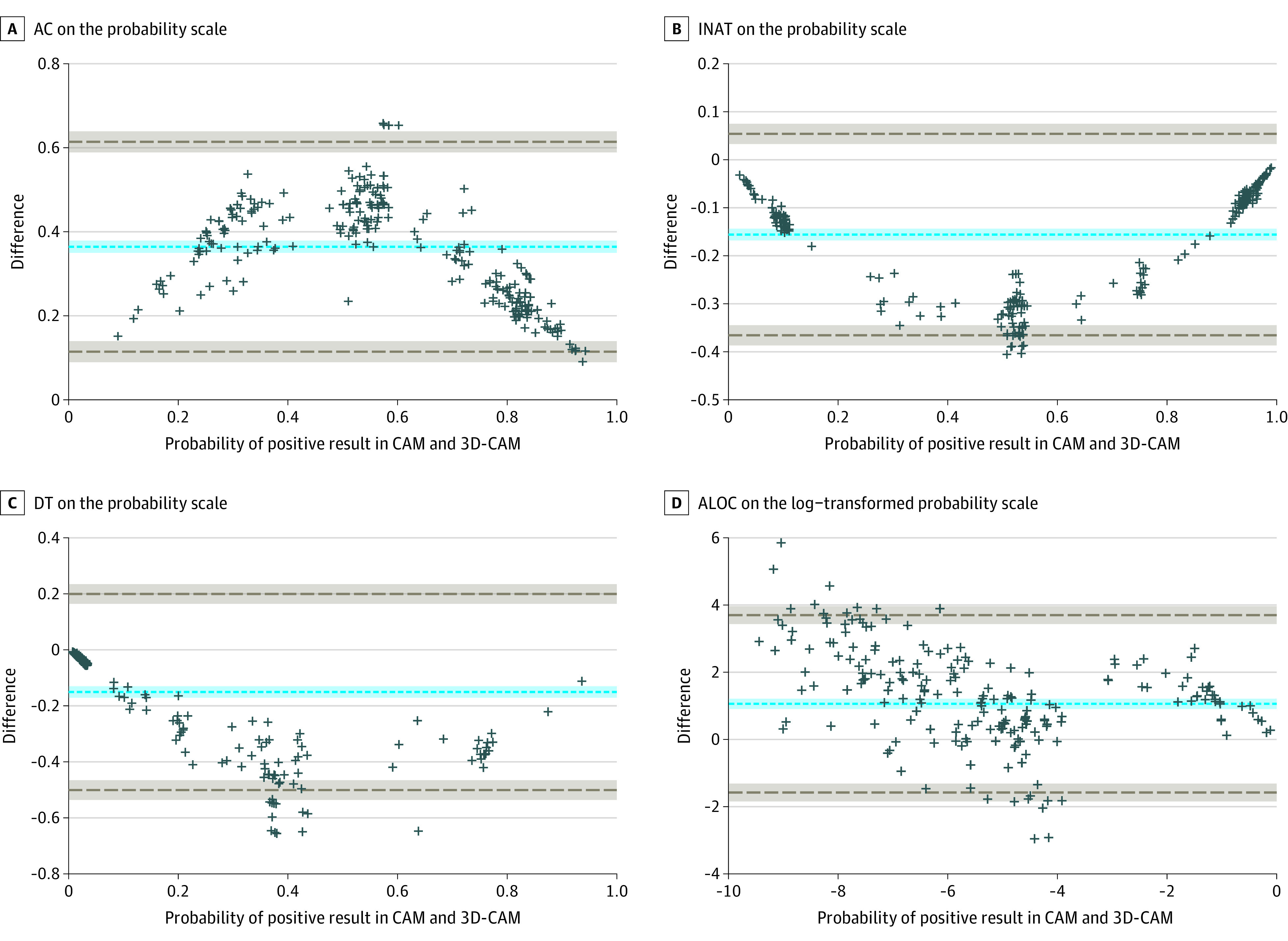
Bland-Altman Plots for 4 Components of the 3-Minute Diagnostic Confusion Assessment Method (3D-CAM) and Long-Form CAM Instruments Each plus sign represents 1 patient (299 total patients); dashed gray lines, 95% agreement limits; and the dashed blue line, the mean difference. AC indicates acute change component; ALOC, altered level of consciousness component; DT, disorganized thinking component; INAT, inattention component.

Additional Bland-Altman diagrams were generated for each of the 4 features (Figure 2). One feature, altered level of consciousness, was plotted on a log scale since the data must be normally distributed for Bland-Altman analysis. The mean difference on the probability scale for acute change was 0.36 (95% CI, 0.35 to 0.38), meaning that the CAM was 0.36 more likely to score for acute changes compared with the 3D-CAM. The mean difference for inattention was −0.16 (95% CI, −0.17 to −0.14); ie, the 3D-CAM was 0.16 more likely to score for inattention. The mean difference for disorganized thinking was −0.15 (95% CI, −0.17 to −0.13); the 3D-CAM was 0.15 more likely to score for disorganized thinking. Finally, altered level of consciousness had a mean difference of 1.06 (95% CI, 0.91 to 1.21), meaning the CAM was 2.89 times as likely to score for altered level of consciousness compared with the 3D-CAM.

The Cohen κ with repeated measures for delirium using the 2 instruments for the 299 patients returned a κ value of 0.71 (95% CI, 0.58-0.83). By feature, Cohen κ testing returned values of 0.17 (95% CI, 0.12-0.23) for acute change, 0.57 (95% CI, 0.49-0.65) for inattention, 0.39 (95% CI, 0.26-0.51) for disorganized thinking, and 0.37 (95% CI, 0.13-0.60) for altered level of consciousness.

## Discussion

We compared a research approach (ie, the original CAM instrument) with a brief clinical assessment (3D-CAM). We found that both instruments had high interrater reliability and good overall agreement (κ = 0.71). However, the 3D-CAM tended to have more positive diagnoses for delirium when compared with the long-form CAM. This is not unexpected given that the 3D-CAM was designed to have high sensitivity as a screening instrument, so cases of delirium would not be missed; therefore, there may be false positives. In clinical practice, a brief screening test would be followed by a longer confirmatory process by a clinician.

The reference standard used in this study, the CAM, has been found to be a reliable assessment tool, and has been validated against standard psychiatry interview and the *Diagnostic and Statistical Manual of Mental Disorders* (Fourth Edition) (*DSM-IV*) and *DSM-IV-TR* criteria in multiple studies.^7,19^ The CAM has also demonstrated excellent psychometric properties in detecting hypoactive delirium that often goes undiagnosed in a clinical setting.^20^ The use of the long CAM approach presented in this study is primarily intended for research application and does present barriers for clinical use, including training requirements and the time to administer and score the instrument. In clinical practice, the shorter CAM is often scored using a brief cognitive screener, such as the Mini-Cog screening instrument or the Short Portable Mental Status Questionnaire, which yields a highly sensitive and quick approach.

Previously, other brief delirium assessments have been presented and validated in various patient populations.^21,22,23,24^ Another derivation of the CAM, the CAM for the Intensive Care Unit (CAM-ICU), was developed to identify delirium in a high-risk population (ie, ICU patients).^25^ The CAM-ICU was derived and is targeted at patients who are unable to speak (eg, are intubated or have a tracheostomy) and agrees well with standard psychiatrist interviews in those populations. When compared with reference standard interviews with patients who could speak, the CAM-ICU has been found to have a sensitivity of 53% and specificity of 100%, and the 3D-CAM has been found to have a sensitivity of 95% and specificity of 93%.^26^

In the postanesthesia care unit, the CAM-ICU has also been compared with the Nursing Delirium Screening Scale (NuDESC) as well as reference standard interview.^27^ When compared with the reference standard, neither tool was shown to have a sensitivity greater than 32%, but each maintained greater than 92% specificity. In nonsurgical settings, the NuDESC was found to have a sensitivity of 86% and a specificity of 87% when compared with the CAM.^28^ The optimal screening instrument for delirium may vary according to intended use and setting.

### Strengths and Limitations

Our study had notable strengths. In studies testing these assessment methods, it has generally not been possible to conduct simultaneous assessment as we did in the current study, either because of masking requirements or the use of a nonoverlapping questions. Since delirium is a fluctuating disorder, assessments that are not conducted at the same time might be discordant due to the time separation. Therefore, our ability to use the 3D-CAM and CAM concurrently was a methodological strength that allowed us to evaluate the instruments without considering the confounding effect of delirium’s fluctuating course. Other strengths with our approach included rigorous training protocols, different statistical methods with generally concordant findings, and results with high precision (ie, narrow confidence intervals).

This study also had several important limitations. We did not conduct what is commonly referred to as a reference standard structured interview by an experienced physician-rater, as is common in other validation studies, and instead compared the 3D-CAM with the CAM. This limitation is difficult to overcome because there is no objective criterion standard for delirium diagnosis (such as a clinical biomarker), and the notion that expert clinicians provide a reference standard is regarded as controversial. Nonetheless, it is not surprising that there would be substantial overlap between 2 instruments that have many assessment questions in common. It is also possible that the 3D-CAM had false positives because it was designed as a highly sensitive instrument, while the long-form CAM had false negatives because it was designed for research, or that results included a combination of both. Ultimately, these questions cannot be resolved without an objectively calibrated reference standard, which for delirium does not currently exist. There is potential that, because assessors were trained in both the CAM and 3D-CAM, CAM assessors could have determined the outcome of the 3D-CAM instrument and biased their scoring; however, all assessments were reviewed by a third party for accurate instrument scoring. Also, scoring was not done during questioning, so it is unlikely that CAM assessors would have known the outcome of the 3D-CAM in real time.

The data were collected from a convenience sample of patients at a single center, which might not be generalizable to other patients or institutions. Additionally, although the time for conducting the interviews was recorded, the time spent scoring each instrument was not noted. The 3D-CAM is much briefer than the long CAM approach, and thus, may be more readily applicable in the clinical setting. The results of this study might not generalize to non-postoperative settings because features of delirium such as altered level of consciousness might be different in postoperative settings. Assessment of an instrument’s performance should not be based on a single study, and other studies should refine these findings in determining the utility and accuracy of the 3D-CAM in patients who have undergone surgery. Although the results seem to suggest that the 3D CAM overdiagnoses delirium, it is also possible that the long CAM underdiagnoses delirium, or, as has previously been noted, that some of the apparent false-positive 3D-CAM diagnoses are actually indicative of subsyndromal delirium.^8^ Finally, to maintain masking, the ordering of the 2 instruments was the same at each assessment time point and may have affected the performance characteristics because of ordering effects, even though the items in the cognitive assessment were the same.

## Conclusions

It might reasonably be concluded that the best tool for screening for delirium depends on the target patient population and context. The CAM and 3D-CAM are unsuitable for patients who cannot speak, making the CAM-ICU a more appropriate tool in this circumstance. The CAM and the 3D-CAM, on the other hand, are likely to be more appropriate than the CAM-ICU on postsurgical wards, where patients tend to be able to speak. In addition, the CAM and the 3D-CAM provide a structured interview and scoring system with excellent interrater reliability. Overall, the CAM is likely the most reliable of these 3 instruments based on extensive testing in multiple clinical contexts,^29^ and the long-form CAM is currently the best validated for research purposes. The 3D-CAM takes less than 3 minutes to complete and would be more suitable for clinical application. Given the possibility of false positives that exists with any highly sensitive screening measure, it is recommended that the diagnosis be confirmed with a more established method, such as the long-form CAM or by *DSM-5* criteria.
